# Bitter-sweet? The role of glycemic control in breast reduction surgery

**DOI:** 10.1186/s12893-026-03507-w

**Published:** 2026-01-21

**Authors:** Samuel Knoedler, Thomas Schaschinger, Felix J. Klimitz, Victoria Kong, Julius M. Wirtz, Omar Allam, Fabio O. Marcela, Jun Jiang, Gabriel Hundeshagen, Adriana C. Panayi, Fortunay Diatta, Martin Kauke-Navarro

**Affiliations:** 1 Department of Plastic Surgery and Hand Surgery, Klinikum Rechts der Isar, Technical University of Munich, Munich, Germany; 2 Department of Surgery, Division of Plastic and Reconstructive Surgery, Yale School of Medicine, Yale New Haven Hospital, New Haven, CT USA; 3 Department of Hand-, Plastic and Reconstructive Surgery, Microsurgery, Burn Trauma Center, BG Trauma Center Ludwigshafen, University of Heidelberg, Ludwigshafen, Germany; 4 Faculty of Medicine, University of Freiburg, Freiburg, Germany; 5 Department of Diagnostic and Interventional Radiology, University Hospital Frankfurt, Frankfurt am Main, Germany; 6 Department of Oral and Maxillofacial Surgery, Charité – Universitätsmedizin Berlin, corporate member of Freie Universität Berlin and Humboldt-Universität zu Berlin, Berlin, Germany

**Keywords:** Breast reduction, Reduction mammoplasty, Diabetes, Diabetes mellitus, Insulin, NSQIP

## Abstract

**Background:**

The American Society of Plastic Surgeons guidelines emphasize the need for additional evidence regarding perioperative glycemic control in patients with diabetes undergoing breast reduction surgery. This study evaluates the association between preoperative hemoglobin A1c (HbA_1c_) levels and postoperative complications in patients with diabetes undergoing reduction mammaplasty.

**Methods:**

A retrospective cohort study of the National Surgical Quality Improvement Program (NSQIP) database was performed, identifying patients diagnosed with diabetes who underwent breast reduction surgery from 2021 to 2023. Patients were stratified by preoperative HbA_1c_ levels: well-controlled (HbA_1c_ < 6.5%) versus poorly controlled (HbA_1c_ ≥ 6.5%). Demographics, surgical characteristics, and 30-day postoperative outcomes were compared between groups. Multivariable logistic regression analysis was performed to identify independent risk factors for complications.

**Results:**

A total of 364 female patients with diabetes met the inclusion criteria, of whom 293 (80%) had non–insulin-dependent and 71 (20%) had insulin-dependent diabetes. Based on HbA_1c_ levels, 206 patients (57%) had well-controlled diabetes, and 158 (43%) had poorly controlled diabetes. The groups were comparable in age, BMI, and surgical characteristics. The overall complication rate was 10.7% (*n* = 39), with superficial surgical site infections being the most common (*n* = 22, 6.0%). Patients with poorly controlled diabetes had higher rates complications overall (13.0% vs. 9.2%, *p* = 0.29) and readmission (4.4% vs. 1.0%, *p* = 0.044). In multivariable analysis, HbA_1c_ ≥ 6.5% was independently associated with an increased risk of complications (OR 2.2, 95% CI 1.0–4.7, *p* = 0.047).

**Conclusion:**

Patients with diabetes and poorly controlled glycemic status demonstrate a higher likelihood of postoperative complications following breast reduction surgery, although the association reached only borderline statistical significance. These findings support the clinical plausibility that suboptimal glycemic control may contribute to increased perioperative risk and may help inform patient counseling and surgical decision-making in this population. Further research is needed to clarify the extent to which preoperative glycemic optimization influences outcomes in this population.

**Supplementary Information:**

The online version contains supplementary material available at 10.1186/s12893-026-03507-w.

## Introduction

Reduction mammaplasty ranks among the most frequently performed procedures in plastic and reconstructive surgery, offering substantial symptomatic relief for patients suffering from macromastia while concurrently delivering significant improvements in quality of life [1–3]. As surgical techniques advance and patient eligibility expands, ensuring optimal perioperative outcomes has become imperative—particularly in the context of an aging and increasingly comorbid patient population [4].

Diabetes mellitus (DM) is among the comorbidities of particular concern in contemporary surgical practice [5, 6]. The global burden of DM has reached epidemic proportions, with research projecting that by 2030, nearly one in six patients undergoing surgery will be affected by some form of the disease [4]. This rising prevalence has important implications for surgical safety and resource allocation. Glycated hemoglobin (HbA_1c_) has emerged as the cornerstone biomarker for assessing long-term glycemic control, reflecting mean blood glucose levels over an approximately three-month period [7]. Its predictive utility in stratifying perioperative risk across numerous surgical disciplines has been well-established in the literature [8, 9]. Diabetes has also been consistently linked with higher postoperative morbidity, including increased rates of infection, delayed wound healing, hematoma or seroma formation, and prolonged hospitalization [10, 11]. Despite this extensive body of evidence, there remains a surprising paucity of data specifically addressing the role of diabetes and glycemic control in reduction mammaplasty. The American Society of Plastic Surgeons (ASPS), in its most recent Clinical Practice Guideline for Reduction Mammaplasty, has formally acknowledged this void in evidence [12]. The guideline explicitly underscores the need for additional research, stating that “[...] research is necessary to further delineate the relative risks of diabetes mellitus [...]” and that “[f]urther studies evaluating the significance of diabetes mellitus as a risk factor would be an important contribution to this body of research.” [12] This call to action highlights the urgency and clinical relevance of expanding the evidence base in this field.

In response to this recognized knowledge gap, our research group previously conducted a large-scale analysis using multi-institutional data, demonstrating that while patients with non-insulin-dependent diabetes mellitus (NIDDM) did not exhibit significantly increased risk, those with insulin-dependent diabetes mellitus (IDDM) experienced markedly higher complication rates following breast reduction surgery [13]. These findings prompted the need for a more nuanced investigation into the role of glycemic control itself—beyond the binary classification of diabetes status—as a determinant of surgical outcomes. To address this knowledge gap, we conducted a retrospective analysis using multi-institutional data from the American College of Surgeons (ACS) National Surgical Quality Improvement Program (NSQIP), a widely utilized outcomes registry in surgical research [14–21].

The present study aims to explore the association between preoperative HbA_1c_ levels and 30-day postoperative outcomes in patients undergoing reduction mammaplasty. By investigating the prognostic relevance of HbA_1c_ levels in this context, our study seeks to address a critical gap in the current literature and to provide data-driven guidance for preoperative risk assessment and perioperative management. These findings may ultimately contribute to more personalized surgical planning, refined patient selection, and improved safety profiles in an increasingly complex patient cohort. Moreover, they have the potential to inform future clinical guidelines and support the development of evidence-based protocols for glycemic optimization in elective plastic surgery.

## Methods

### Data source

We utilized data from the ACS-NSQIP, a well-validated, risk-adjusted, outcomes-based initiative designed to evaluate and improve the quality of surgical care across nearly 700 participating institutions in more than ten countries. The database captures standardized information on over 150 variables, including detailed preoperative risk factors, intraoperative characteristics, and 30-day postoperative outcomes. Data abstraction is carried out by trained surgical clinical reviewers at each site using uniform definitions and protocols, thereby ensuring a high level of accuracy, reproducibility, and inter-institutional comparability. All patient data are fully deidentified, and as such, this study was deemed exempt from human subjects research regulations by the Yale School of Medicine Institutional Review Board (Protocol ID: 2000035387).

### Patient selection

We analyzed data from the ACS-NSQIP database for the years 2021 through 2023, the only period during which HbA_1c_ values were systematically recorded. The ACS-NSQIP database exclusively captures surgical cases involving patients aged 18 years and older; therefore, non-surgical cases as well as pediatric and adolescent patients were not included in this study.

We identified patients who underwent bilateral reduction mammaplasty using the Current Procedural Terminology (CPT) code 19,318. To ensure appropriate procedural indication, cases were limited to those associated with International Classification of Diseases (ICD)-9-Clinical Modification (CM) codes 611.1 (hypertrophy of breast) or V50.1 (other plastic surgery for unacceptable cosmetic appearance), and ICD-10-CM codes N62 (hypertrophy of breast) or Z41.1 (other plastic surgery for unacceptable cosmetic appearance). Only adult female patients with a documented diagnosis of diabetes mellitus and a recorded HbA_1c_ value were included. We excluded (i) all patients whose gender was recorded as male, non-binary, or transgender, based on demographic and diagnosis entries, (ii) patients classified as American Society of Anesthesiologists (ASA) Physical Status Classification IV, (iii) cases performed under any form of anesthesia other than general anesthesia, and (iv) any patients with missing or unrecorded diabetes status. To ensure procedural homogeneity, minimize potential miscoding, and limit variability arising from differences in surgical training operative technique, we further excluded (v) patients who underwent any additional invasive procedures besides breast reduction, (vi) cases associated with neoplastic diagnoses—both benign and malignant, and (vii) any procedures performed outside of the general or plastic surgery specialties. Supplementary Table 1 provides an overview of the inclusion and exclusion criteria. This selection process resulted in a well-defined cohort of adult women with diabetes who underwent breast reduction and had documented preoperative HbA_1c_ values.

### Data extraction

All variables were defined and recorded according to standardized ACS-NSQIP criteria, as outlined in the ACS-NSQIP Participant Use Data File (PUF) User Guide, and were consistently available across all years included in the study period [22, 23]. We analyzed detailed preoperative demographic and clinical information, including age, self-reported racial identity, and body mass index (BMI). BMI was calculated using the standard formula [weight (kg)/height (m²)] and categorized according to World Health Organization (WHO) guidelines: underweight (< 18.5 kg/m²), normal weight (18.5–24.9 kg/m²), overweight (25.0–29.9 kg/m²), and obese (≥ 30.0 kg/m²). Diabetes mellitus status was stratified into NIDDM and IDDM. A complete list of preoperative variables is provided in Table 1.

**Table 1:** Demographics and preoperative health characteristics of all patients. Reported as n (%), unless otherwise stated

Patient Characteristics	All (*n* = 364)		HbA_1c_ < 6.5% (*n* = 206)		HbA_1c_ ≥ 6.5% (*n* = 158)		*p*
Preoperative HbA_1c_ [%]	6.5	± 1.2	5.7	± 0.5	7.5	± 1.2	< 0.001
Age, mean ± SD [years]	51	± 13	51	± 12	52	± 14	0.11
BMI, mean ± SD [kg/m^2^]	34	± 7.4	34	± 8.3	34	± 6.1	0.42
BMI Class							0.79
Underweight	7	(1.9)	5	(2.4)	2	(1.3)	
Normal Weight	13	(3.6)	7	(3.4)	6	(3.8)	
Overweight	66	(18)	35	(17)	31	(20)	
Obesity	278	(76)	159	(77)	119	(75)	
Race							0.30
White	131	(36)	79	(38)	52	(33)	
Black or African American	143	(39)	77	(37)	66	(42)	
Asian	10	(2.7)	4	(1.9)	6	(3.8)	
American Indian or Alaska Native	2	(0.6)	0	(0.0)	2	(1.3)	
Native Hawaiian or Other Pacific Islander	2	(0.6)	1	(0.5)	1	(0.6)	
Diabetes							< 0.001
Non-Insulin	293	(80)	186	(90)	107	(68)	
Insulin	71	(20)	20	(9.7)	51	(32)	
Smoking	23	(6.3)	12	(5.8)	11	(7.0)	0.66
COPD	6	(1.6)	1	(0.5)	5	(3.2)	0.089
CHF	12	(3.3)	3	(1.5)	9	(5.7)	0.036
Dialysis	4	(1.1)	2	(1.0)	2	(1.3)	> 0.99
Hypertension	213	(59)	109	(53)	104	(66)	0.013
Renal Failure	0	(0.0)	0	(0.0)	0	(0.0)	> 0.99
Ascites	0	(0.0)	0	(0.0)	0	(0.0)	> 0.99
Corticosteroid Use	23	(6.3)	16	(7.8)	7	(4.4)	0.28
Disseminated Cancer	0	(0.0)	0	(0.0)	0	(0.0)	> 0.99
Bleeding Disorder	4	(1.1)	3	(1.5)	1	(0.6)	0.64
Preoperative Transfusions	0	(0.0)	0	(0.0)	0	(0.0)	> 0.99
Functional Status							0.43
Independent	360	(99)	205	(100)	155	(98)	
Partially Dependent	1	(0.3)	0	(0.0)	1	(0.6)	
ASA Class							0.36
1 - No Disturbance	4	(1.1)	3	(1.5)	1	(0.6 )	
2 - Mild disturbance	185	(51)	110	(53)	75	(47)	
3 - Severe Disturbance	175	(48)	93	(45)	82	(52)	

The primary exposure variable was the preoperative HbA_1c_ level, expressed as a percentage, which was analyzed both as a continuous variable and dichotomized using the clinically accepted threshold of 6.5%. Patients were categorized into two groups: well-controlled diabetes (HbA_1c_ < 6.5%) and poorly controlled diabetes (HbA_1c_ ≥ 6.5%). This cut-off was selected in accordance with American Diabetes Association (ADA) guidelines and reflects current standards in perioperative risk stratification across surgical disciplines [24–26].

Perioperative characteristics included operative time, length of hospital stay, surgical specialty, surgical setting, and year of surgery (Table 2). The primary postoperative outcome was a composite endpoint, “any complication,” defined as the occurrence of at least one ACS-NSQIP–defined adverse event within 30 days postoperatively, including mortality, unplanned readmission, surgical complications, or medical complications. An overview of all postoperative outcomes is presented in Table 3.

**Table 2:** Surgical characteristics of all patients. Reported as n (%), unless otherwise stated

Surgical Characteristics	All (*n* = 364)		HbA_1c_ < 6.5% (*n* = 206)		HbA_1c_ ≥ 6.5% (*n* = 158)		*p*
Operative time, mean ± SD [min]	155	± 63	155	± 60	155	± 67	0.62
Length of Hospital Stay, mean ± SD [days]	0.32	± 2.1	0.22	± 0.61	0.46	± 3.1	0.95
Surgical Specialty							> 0.99
General Surgery	5	(1.4)	3	(1.5)	2	(1.3)	
Plastic Surgery	359	(99)	203	(99)	156	(99)	
Setting							0.46
Inpatient	17	(4.7)	8	(3.9)	9	(5.7)	
Outpatient	347	(95)	198	(96)	149	(94)	
Year							0.30
2021	84	(23)	48	(23)	36	(23)	
2022	123	(34)	63	(31)	60	(38)	
2023	157	(43)	95	(46)	62	(39)	

**Table 3:** Postoperative outcomes of all patients. Reported as n (%), unless otherwise stated

Outcomes	All (*n* = 364)		HbA_1c_ < 6.5% (*n* = 206)		HbA_1c_ ≥ 6.5% (*n* = 158)		*p*
Any Complication	39	(11)	19	(9.2)	20	(13)	0.29
Mortality	0	(0.0)	0	(0.0)	0	(0.0)	> 0.99
Reoperation	6	(1.6)	4	(1.9)	2	(1.3)	0.70
Readmission	9	(2.5)	2	(1.0)	7	(4.4)	**0.044**
Surgical Complication	28	(7.7)	15	(7.3)	13	(8.2)	0.74
Superficial incisional infection	22	(6.0)	11	(5.3)	11	(7.0)	0.52
Deep incisional infection	1	(0.3)	0	(0.0)	1	(0.6)	0.43
Organ space infection	1	(0.3)	1	(0.5)	0	(0.0)	> 0.99
Dehiscence	2	(0.5)	2	(1.0)	0	(0.0)	0.51
Bleeding Requiring Transfusion	3	(0.8)	1	(0.5)	2	(1.3)	0.58
Medical Complication	3	(0.8)	1	(0.5)	2	(1.3)	0.58
Pneumonia	0	(0.0)	0	(0.0)	0	(0.0)	> 0.99
Unplanned intubation	0	(0.0)	0	(0.0)	0	(0.0)	> 0.99
Ventilator > 48 h	0	(0.0)	0	(0.0)	0	(0.0)	> 0.99
Acute Renal Failure	0	(0.0)	0	(0.0)	0	(0.0)	> 0.99
Renal Insufficiency	0	(0.0)	0	(0.0)	0	(0.0)	> 0.99
Urinary Tract Infection	2	(0.6)	1	(0.5)	1	(0.6)	> 0.99
Stroke/CVA	0	(0.0)	0	(0.0)	0	(0.0)	> 0.99
Cardiac Arrest Requiring CPR	0	(0.0)	0	(0.0)	0	(0.0)	> 0.99
Pulmonary Embolism	0	(0.0)	0	(0.0)	0	(0.0)	> 0.99
Myocardial Infarction	0	(0.0)	0	(0.0)	0	(0.0)	> 0.99
DVT Requiring Therapy	0	(0.0)	0	(0.0)	0	(0.0)	> 0.99
Sepsis	1	(0.3)	0	(0.0)	1	(0.6)	0.43
Septic Shock	0	(0.0)	0	(0.0)	0	(0.0)	> 0.99
Discharge Destination							0.43
Home	363	(100)	206	(100)	157	(99)	
Facility Not Home	1	(0.3)	0	(0.0)	1	(0.6)	

*CVA* Cerebrovascular Accident, *CPR* Cardio Pulmonary Resuscitation, *DVT * Deep Vein Thrombosis

### Statistical analysis

Data from the ACS-NSQIP datasets were initially exported to Microsoft Excel (Version 16, Microsoft Corporation, Redmond, WA, USA) using IBM SPSS Statistics for Windows (Version 29, IBM Corporation, Armonk, NY, USA). The datasets were reformatted to maintain consistent variable structure. Data storage and management were handled using LabArchives, an electronic laboratory notebook platform (LabArchives, LLC, San Marcos, CA, USA). Statistical analyses were conducted with GraphPad Prism (Version 9.00 for MacOS, GraphPad Software, La Jolla, CA, USA). Continuous variables such as age and BMI were analyzed using independent t-tests when normally distributed, with results reported as means and standard deviations. Categorical variables were assessed using Pearson’s chi-squared test, and Fisher’s exact test was applied when expected cell counts were below 10. A p-value of less than 0.05 was considered statistically significant. To evaluate the influence of HbA_1c_ on postoperative outcomes, multivariable logistic regression was conducted to adjust for potential confounding factors. Covariates included in the model were BMI, operative time, length of hospital stay, smoking status, presence of a bleeding disorder, dialysis, medically treated hypertension, corticosteroid use, ASA classification, and surgical setting (inpatient vs. outpatient), when a meaningful or clinically plausible association with the outcomes was suggested by the univariable analysis results (Supplementary Table 2), and when their inclusion did not result in issues of model instability, such as singular matrix.

## Results

### Preoperative patient demographics and health characteristics

A total of 364 female patients with diabetes met inclusion criteria. The mean age was 51 ± 13 years, and the mean BMI was 34 ± 7.4 kg/m². Based on preoperative HbA_1c_ levels, 206 patients (57%) had well-controlled diabetes (HbA_1c_ < 6.5%), while 158 patients (43%) had poorly controlled diabetes (HbA_1c_ ≥ 6.5%). The mean HbA_1c_ was 5.7 ± 0.51% in the well-controlled group and 7.5 ± 1.2% in the poorly controlled group (*p* < 0.001). The two groups were comparable in terms of age and BMI. However, poorly controlled patients exhibited significantly higher rates of insulin dependence (32% vs. 10%, *p* < 0.001), hypertension (66% vs. 53%, *p* = 0.013), and congestive heart failure (5.7% vs. 1.5%, *p* = 0.036). Additional baseline characteristics are summarized in Table 1.

### Surgical characteristics

Both groups showed similar operative times (155 ± 60–67 min, *p* = 0.62) and hospital stays (0.2 ± 0.6 days vs. 0.5 ± 3.1 days, *p* = 0.95). The majority of procedures (*n* = 347, 95%) were performed in an outpatient setting. Case distribution remained consistent throughout the study period (2021–2023). Further details are presented in Table 2.

### Postoperative outcomes

**Fig. 1:**
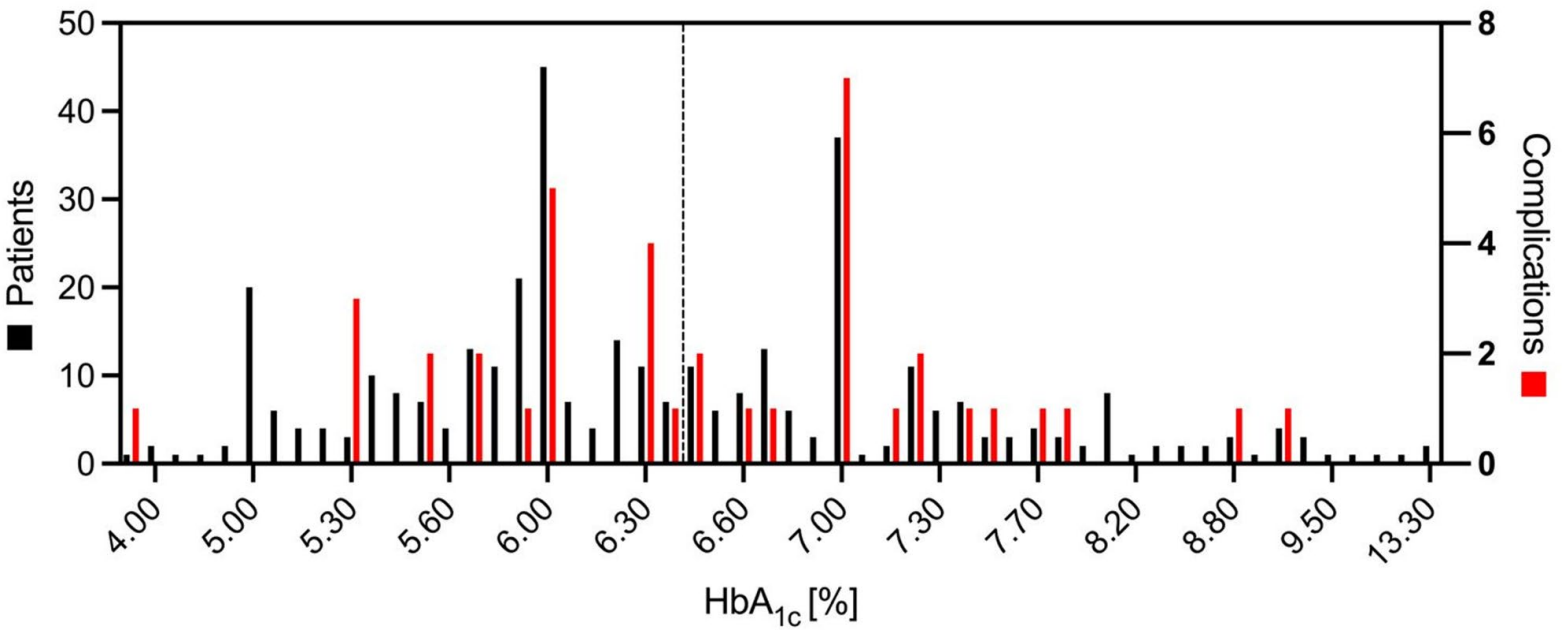
Distribution of patients and complications by HbA₁c levels. Figure 1 shows the number of patients (black bars, left y-axis) and the number of postoperative complications (red bars, right y-axis) stratified by preoperative HbA₁c [%]. Each bar represents a discrete HbA₁c value. The dashed vertical line marks the threshold of HbA₁c = 6.5%. Note that the number of complications may exceed the number of patients in a given HbA₁c category, as a single patient may experience multiple complications

The overall 30-day complication rate was 10.7% (*n* = 39). Patients with poorly controlled diabetes experienced a higher, though not statistically significant, rate of any complication compared to those with well-controlled diabetes (13% vs. 9.2%, *p* = 0.29). Unplanned readmissions were significantly more frequent in the poorly controlled group (4.4% vs. 1.0%, *p* = 0.044). Reoperation rates were comparable (1.3% vs. 1.9%, *p* = 0.70), and no perioperative mortality occurred. The most common surgical complication was superficial incisional infection (6.0%, *n* = 22), with slightly higher rates observed in the poorly controlled group (7.0% vs. 5.3%, p *= 0.52*). Other surgical complications—including deep incisional infections, wound dehiscence, and bleeding requiring transfusion—were infrequent in both cohorts. Medical complications occurred in 0.8% of patients overall, with slightly higher rates among poorly controlled patients (1.3% vs. 0.5%, *p* = 0.58). No cases of major medical complications (e.g., pneumonia, cardiovascular events, or thromboembolic events) were observed. Full outcome data are provided in Table 3; Fig. 1.

### Multivariable analysis

After adjusting for potential confounding variables, poorly controlled glycemic status (HbA_1c_ ≥ 6.5%) emerged as an independent predictor of postoperative complications. Patients with HbA_1c_ ≥ 6.5% had significantly increased odds of experiencing any complication (OR 2.2, 95% CI 1.0-4.7, *p* = 0.047) compared to those with well-controlled diabetes. The odds ratio for surgical complications was 2.1 (95% CI 0.85–5.1, *p* = 0.11), and for medical complications, 2.9 (95% CI 0.25–33, *p* = 0.40). The wide confidence intervals for medical complications reflect the low number of observed events. The multivariable model demonstrated good discriminative ability and confirmed that HbA_1c_ ≥ 6.5% represents a clinically meaningful threshold for risk stratification in patients with diabetes undergoing breast reduction surgery. Complete results of the multivariable analysis are presented in Table 4.

**Table 4:** Univariable and multivariable analysis of HbA1c ≥ 6.5% in complications

	Multivariable Analysis			Univariable Analysis		
	OR	CI (95%)	*P*	OR	CI (95%)	*P*
Any Complication	2.2	(1.0–4.7)	**0.047**	1.4	(0.74–2.8)	0.29
Surgical Complication	2.1	(0.85–5.1)	0.11	1.2	(0.54–2.5)	0.74
Medical Complication	2.9	(0.25–33)	0.40	2.2	(0.29–17)	0.58

Statistically significant *p* values are highlighted in bold

## Discussion

This study revealed that patients with diagnosed diabetes and poorly controlled glycemic status (HbA_1c_ ≥ 6.5%) had a higher likelihood of postoperative complications following breast reduction surgery. Although the association reached borderline statistical significance (*p* = 0.047), the absolute difference in complication rates of 13.0% in patients with poorly controlled diabetes versus 9.2% in those with well-controlled diabetes represents a clinically meaningful increase. Importantly, our finding that HbA_1c_ ≥ 6.5% independently predicts a 2.2-fold increased risk of any complication provides procedure-specific evidence that may help address a key evidence gap identified in current ASPS guidelines, which may ultimately help guide surgical decision-making in this growing patient population.

The overall complication rate of 10.7% in our cohort of patients diagnosed with diabetes aligns with previous NSQIP studies of breast reduction surgery. Fischer et al. reported an overall complication rate of 5.1% in their analysis of 3,538 breast reduction patients from 2005 to 2010 NSQIP data [27], while Hanwright et al. found a 4.47% complication rate in 2,507 reduction mammaplasty patients from 2006 to 2010 [28]. Our higher complication rate may reflect the inherent increased risk associated with diabetes, as suggested by previous studies demonstrating elevated morbidity in patients with diagnosed diabetes undergoing various plastic surgery procedures [29]. Previous analysis of nearly 40,000 plastic surgery patients found that insulin-dependent patients with diabetes had a 2.05-fold increased risk of wound dehiscence and a 1.55-fold increased risk of wound infection compared to patients without diagnosed diabetes [30]. Our findings extend this evidence to breast reduction surgery and suggest that an HbA1c ≥ 6.5% may serve as an early risk indicator, though not necessarily a strict causal determinant.

Chronic hyperglycemia impairs vascular function, angiogenesis, keratinocyte migration, fibroblast activity, and immune responses, collectively delaying bacterial clearance and prolonging inflammation [31–35]. In our cohort, this pathophysiology manifested primarily as wound-healing complications, consistent with an operation in which large soft-tissue flaps depend heavily on reliable perfusion and rapid epithelialization. The observation that risk begins to rise at an HbA1c level of 6.5% is also consistent with literature across other surgical fields, where even modest elevations are associated with higher surgical site infection and complication rates [8, 36, 37]. Our study suggests that breast reduction surgery follows a similar trajectory, as we observed increased complications at 6.5% threshold (Fig. 1).

Clinically, the observed absolute complication difference of approximately 4% and the 2.2-fold adjusted odds ratio may help surgeons in risk stratification and shared decision-making. This study provides procedure-specific evidence to support the use of HbA1c in preoperative risk assessment. As current guidelines provide limited specialty-specific direction on perioperative glycemic management, our results support incorporating HbA1c into preoperative pathways for breast reduction patients with diabetes rather than relying on diabetes status or insulin dependence alone.

The significantly higher readmission rate observed in patients with poor glycemic control (4.4% vs. 1.0%) represents a clinically and economically important finding. Although causality cannot be inferred, this pattern suggests that suboptimal glycemic control may contribute to preventable postoperative healthcare utilization. Surgical site infections accounted for most complications in this cohort, frequently presenting after discharge and being strongly associated with unplanned readmissions. Readmissions are a major driver of postoperative costs across multiple surgical fields [38]. Furthermore, orthopedic studies have specifically linked elevated HbA_1c_ to increased same-day and 90-day expenditures [39, 40]. Similarly, our findings indicate that poorly controlled glycemia may drive preventable resource utilization in elective plastic surgery. In a value-based environment, preoperative HbA_1c_ screening and optimization may serve as a low-cost, high-impact intervention to reduce postoperative morbidity and associated expenditures in patients with diabetes seeking breast reduction surgery. For surgeons, these findings offer actionable guidance on risk stratification and patient optimization. For patients, improved preoperative glycemic control represents an attainable goal that may reduce their risk of adverse outcomes and hospital readmission.

Taken together, our data underscore the need for formal perioperative glycemic management protocols in breast reduction surgery. While randomized prospective trials involving patients with inadequately controlled diabetes would be ethically inappropriate, prospective observational studies are needed to refine perioperative glycemic optimization. Future research should aim to identify clinically meaningful HbA1c thresholds and to determine how long adequate glycemic control must be sustained before surgery can be performed safely. Additionally, evaluating the feasibility and cost-effectiveness of structured preoperative glycemic optimization programs may help inform institutional guidelines. Ultimately, our findings highlight an important and modifiable risk factor that can be leveraged by patients, surgeons, and healthcare systems to enhance surgical outcomes and resource utilization in breast reduction surgery.

## Limitations

This study has several important limitations that should be considered when interpreting the results. First, the retrospective nature of NSQIP data analysis limits our ability to establish a causal relationship between elevated HbA_1c_ levels and postoperative complications. The NSQIP database also provides only 30-day postoperative follow-up, which may underestimate true complication rates, particularly for wound healing issues that often manifest beyond the first postoperative month. Second, the timing of preoperative HbA1c measurement was not consistently available for all cases and likely varied among patients. Because HbA1c reflects glycemic control over the preceding 2–3 months, measurements obtained at different intervals prior to surgery may not correspond precisely to perioperative metabolic status. Additionally, NSQIP does not provide information regarding modifications in diabetes management—such as initiation or adjustment of oral agents or insulin—between HbA1c assessment and the operative date. The database captures only a single preoperative HbA1c measurement, and it does not provide information on whether glycemic regulation was maintained during the postoperative period. Consequently, we cannot determine whether patients with well-controlled HbA1c at the time of surgery had fluctuating glucose levels before or after the procedure, nor can we assess the impact of sustained versus transient metabolic control. These unmeasured variables may influence perioperative glycemia and thus represent potential sources of residual confounding. Third, the sample size of 364 patients with diagnosed diabetes, while adequate to detect overall differences, limits more detailed subgroup analyses. Fourth, further potentially important confounders were not captured in the NSQIP database. These include duration and severity of diabetes, use of specific antidiabetic medications such as insulin or GLP-1 receptor agonists, the presence of diabetes-related end-organ damage, and perioperative glucose management protocols. Any of these factors could influence both HbA_1c_ levels and surgical risk, and their omission may affect the observed associations. Finally, the reliance on NSQIP data, while providing standardized collection methods, may not represent all practice patterns, and the binary HbA_1c_ threshold of 6.5%, while clinically relevant, may not represent the optimal cutoff for surgical risk stratification.

## Conclusion

In this study, we demonstrate that poorly controlled glycemic status, defined as HbA_1c_ ≥ 6.5%, is independently associated with an increased risk of postoperative complications following breast reduction surgery. Patients with elevated HbA_1c_ also experienced higher rates of hospital readmission, further underscoring the clinical and economic impact of inadequate preoperative glycemic control. These findings identify HbA_1c_ as a meaningful and potentially modifiable risk factor, particularly relevant in the context of elective procedures. Incorporating HbA_1c_ screening into routine preoperative assessment may aid in risk stratification and improve surgical outcomes. Moreover, targeted glycemic optimization could reduce healthcare utilization and support value-based care initiatives. Prospective observational studies are warranted to refine our understanding of perioperative glycemic optimization. Specifically, future research should examine how long HbA1c levels must be adequately regulated before elective breast reduction can be performed safely, and whether additional biomarkers or short-term measures of glycemic variability improve risk stratification.

## Supplementary Information

Supplementary Material 1.

Supplementary Material 2.

## Data Availability

Datasets generated and analyzed to provide the findings in this study are available from the corresponding author upon reasonable request. The data are not publicly available due to restrictions from the American College of Surgeons National Surgical Quality Improvement Program.
