# Supplementary material for: Bitter-sweet? The role of glycemic control in breast reduction surgery
Source: BMC Surg. 2026 Jan 21;26:79. doi: 10.1186/s12893-026-03507-w (PMC12849521; doi:10.1186/s12893-026-03507-w)
Supplement: Supplementary file 2 — Supplementary Material 2. [file 12893_2026_3507_MOESM2_ESM.docx]

**SUPPLEMENTARY MATERIAL**

**Supplementary Table 1**. Inclusion and Exclusion Criteria for Study Cohort

|  | **Criteria** |
| --- | --- |
| **Inclusion Criteria** | • Adult patients (≥ 18 years)  • Female gender  • Underwent bilateral reduction mammaplasty (CPT 19318)  • Corresponding ICD-9/10 codes for hypertrophy of breast or unacceptable cosmetic appearance  • Documented diagnosis of diabetes mellitus  • Recorded preoperative HbA1c value |
| **Exclusion Criteria** | • Male, non-binary, or transgender patients  • ASA class IV  • Procedures performed under anesthesia other than general anesthesia  • Missing/unrecorded diabetes status  • Concurrent additional invasive procedures  • Neoplastic diagnoses (benign or malignant)  • Procedures performed outside general or plastic surgery specialties  • Non-surgical or pediatric cases (NSQIP inherently excludes < 18 years) |

| **Supplementary Table 2.** Univariate Analysis of all patients. Reported as n (%), unless otherwise stated. | | | | | | |  |  |  |  |  |  |  |  |  |  |  |  |
| --- | --- | --- | --- | --- | --- | --- | --- | --- | --- | --- | --- | --- | --- | --- | --- | --- | --- | --- |
|  |  | **Any Complication** | | | | |  | **Surgical Complication** | | | | |  | **Medical Complication** | | | | |
| **Continuous Variables** |  | Yes | | No | | *P* |  | Yes | | No | | *P* |  | Yes | | No | | *P* |
|  |  |  |  |  |  |  |  |  |  |  |  |  |  |  |  |  |  |  |
| Age, mean ± SD [years] |  | 52 | ± 16 | 51 | ± 13 | 0.69 |  | 51 | ± 17 | 51 | ± 13 | 0.86 |  | 66 | ± 5.0 | 51 | ± 13 | 0.032 |
| Weight, mean ± SD [lbs] |  | 64 | ± 2.4 | 63 | ± 2.9 | 0.045 |  | 64 | ± 2.6 | 63 | ± 2.9 | 0.17 |  | 65 | ± 2.1 | 63 | ± 2.9 | 0.31 |
| Height, mean ± SD [inch] |  | 208 | ± 39 | 194 | ± 37 | 0.022 |  | 214 | ± 40 | 194 | ± 36 | 0.0050 |  | 222 | ± 43 | 196 | ± 37 | 0.24 |
| BMI, mean ± SD [kg/m^2] |  | 36 | ± 6.9 | 34 | ± 7.5 | 0.17 |  | 37 | ± 7.3 | 34 | ± 7.4 | 0.032 |  | 37 | ± 5.3 | 34 | ± 7.4 | 0.34 |
| Sex |  |  |  |  |  | > 0.99 |  |  |  |  |  | > 0.99 |  |  |  |  |  | > 0.99 |
| Female |  | 39 | (11) | 325 | (89) |  |  | 28 | (7.7) | 336 | (92) |  |  | 3 | (0.82) | 361 | (99) |  |
| Race |  |  |  |  |  | 0.97 |  |  |  |  |  | 0.87 |  |  |  |  |  | 0.96 |
| White |  | 14 | (11) | 117 | (89) |  |  | 10 | (7.6) | 121 | (92) |  |  | 2 | (1.5) | 129 | (98) |  |
| Black or African American |  | 14 | (9.8) | 129 | (90) |  |  | 9 | (6.3) | 134 | (94) |  |  | 1 | (0.70) | 142 | (99) |  |
| Asian |  | 1 | (10) | 9 | (90) |  |  | 0 | (0.0) | 10 | (100) |  |  | 0 | (0.0) | 10 | (100) |  |
| American Indian or Alaska Native |  | 0 | (0.0) | 2 | (100) |  |  | 0 | (0.0) | 2 | (100) |  |  | 0 | (0.0) | 2 | (100) |  |
| Native Hawaiian or Other Pacific Islander |  | 0 | (0.0) | 2 | (100) |  |  | 0 | (0.0) | 2 | (100) |  |  | 0 | (0.0) | 2 | (100) |  |
| Diabetes |  |  |  |  |  | 0.15 |  |  |  |  |  | 0.80 |  |  |  |  |  | 0.099 |
| Non-Insulin |  | 28 | (9.6) | 265 | (90) |  |  | 22 | (7.5) | 271 | (92) |  |  | 1 | (0.34) | 292 | (100) |  |
| Insulin |  | 11 | (15) | 60 | (85) |  |  | 6 | (8.5) | 65 | (92) |  |  | 2 | (2.8) | 69 | (97) |  |
| Current Smoker |  | 3 | (13) | 20 | (87) | 0.72 |  | 2 | (8.7) | 21 | (91) | 0.69 |  | 0 | (0.0) | 23 | (100) | > 0.99 |
| COPD |  | 1 | (17) | 5 | (83) | 0.50 |  | 1 | (17) | 5 | (83) | 0.38 |  | 0 | (0.0) | 6 | (100) | > 0.99 |
| CHF |  | 1 | (8.3) | 11 | (92) | > 0.99 |  | 1 | (8.3) | 11 | (92) | > 0.99 |  | 1 | (8.3) | 11 | (92) | 0.096 |
| Dialysis |  | 1 | (25) | 3 | (75) | 0.37 |  | 0 | (0.0) | 4 | (100) | > 0.99 |  | 0 | (0.0) | 4 | (100) | > 0.99 |
| Hypertension |  | 24 | (11) | 189 | (89) | 0.69 |  | 16 | (7.5) | 197 | (92) | 0.88 |  | 3 | (1.4) | 210 | (99) | 0.27 |
| Corticosteroid Use |  | 5 | (22) | 18 | (78) | 0.086 |  | 4 | (17) | 19 | (83) | 0.089 |  | 0 | (0.0) | 23 | (100) | > 0.99 |
| Bleeding Disorder |  | 1 | (25) | 3 | (75) | 0.37 |  | 1 | (25) | 3 | (75) | 0.27 |  | 0 | (0.0) | 4 | (100) | > 0.99 |
| Functional Status |  |  |  |  |  | > 0.99 |  |  |  |  |  | > 0.99 |  |  |  |  |  | > 0.99 |
| Independent |  | 39 | (11) | 321 | (89) |  |  | 28 | (7.8) | 332 | (92) |  |  | 3 | (0.83) | 357 | (99) |  |
| Partially Dependent |  | 0 | (0.0) | 1 | (100) |  |  | 0 | (0.0) | 1 | (100) |  |  | 0 | (0.0) | 1 | (100) |  |
| ASA Class |  |  |  |  |  | 0.62 |  |  |  |  |  | 0.83 |  |  |  |  |  | 0.81 |
| 1 - No Disturbance |  | 0 | (0.0) | 4 | (100) |  |  | 0 | (0.0) | 4 | (100) |  |  | 0 | (0.0) | 4 | (100) |  |
| 2 - Mild disturbance |  | 18 | (9.7) | 167 | (90) |  |  | 14 | (7.6) | 171 | (92) |  |  | 1 | (0.54) | 184 | (99) |  |
| 3 - Severe Disturbance |  | 21 | (12) | 154 | (88) |  |  | 14 | (8.0) | 161 | (92) |  |  | 2 | (1.1) | 173 | (99) |  |
| 4 - Life Threat |  |  |  |  |  |  |  |  |  |  |  |  |  |  |  |  |  |  |
| Operative time, mean ± SD [min] |  | 145 | ± 83 | 156 | ± 60 | 0.031 |  | 150 | ± 95 | 155 | ± 60 | 0.062 |  | 114 | ± 41 | 155 | ± 63 | 0.24 |
| Length of Hospital Stay, mean ± SD [days] |  | 0.21 | ± 0.70 | 0.34 | ± 2.2 | 0.55 |  | 0.25 | ± 0.80 | 0.33 | ± 2.2 | 0.79 |  | 0.0 | ± 0.0 | 0.33 | ± 2.1 | 0.45 |
| Surgical Specialty |  |  |  |  |  | > 0.99 |  |  |  |  |  | > 0.99 |  |  |  |  |  | > 0.99 |
| General Surgery |  | 0 | (0.0) | 5 | (100) |  |  | 0 | (0.0) | 5 | (100) |  |  | 0 | (0.0) | 5 | (100) |  |
| Plastic Surgery |  | 39 | (11) | 320 | (89) |  |  | 28 | (7.8) | 331 | (92) |  |  | 3 | (0.84) | 356 | (99) |  |
| Setting |  |  |  |  |  | 0.41 |  |  |  |  |  | 0.13 |  |  |  |  |  | > 0.99 |
| Inpatient |  | 3 | (18) | 14 | (82) |  |  | 3 | (18) | 14 | (82) |  |  | 0 | (0.0) | 17 | (100) |  |
| Outpatient |  | 36 | (10) | 311 | (90) |  |  | 25 | (7.2) | 322 | (93) |  |  | 3 | (0.86) | 344 | (99) |  |
| SD: Standard Deviation; COPD: Chronic Obstructive Pulmonary Disease; CHF: Congestive Heart Failure; ASA: American Society of Anesthesiologists; | | | | | | | | | | | | | | | | | | |
